# PEGylated nanoceria protect human epidermal cells from reactive oxygen species

**DOI:** 10.1186/1755-8166-7-S1-P78

**Published:** 2014-01-21

**Authors:** Ragini Singh, Ritesh K Shukla, Ashutosh Kumar, Alok Dhawan, Sanjay Singh

**Affiliations:** 1 Institute of Life Sciences, Ahmedabad University, Ahmedabad, Gujarat, India

## Background

Cerium oxide nanoparticles (CeNPs) have shown promise as catalytic antioxidants in cell culture and animal models due to its superoxide dismutase and catalase mimetic activities. CeNPs can exist in +3 and +4 oxidation states, which have been suggested as the mechanism behind the free radical scavenging activity. It has also been shown that unique crystal structure of CeNPs with surface oxygen defects promote the shuffling between the +3 and +4 oxidation states that help in eliminating the free radicals. This activity of CeNPs has been suggested as the mechanism, at least in part, behind increase in cellular longevity and decrease in the toxic insults in mammalian cells/tissues. Further, the uniform distribution of CeNPs, upon cellular uptake, within the cells could prevent the accumulation of reactive oxygen species in the cells.

## Materials and methods

PEG coated CeNPs were synthesized by methods described by Karakoti et al. CeNPs were characterized by UV-visible spectra and dynamic light scattering. Cytotoxicity of CeNPs was done by MTT and Neutral Red Uptake (NRU) assay in a time and dose dependent manner. The level of intracellular ROS generation was estimated by using 2,7-dichlorofluorescein diacetate (DCFDA) dye by time and dose dependent approach. Superoxide dismutase (SOD) mimetic activity of ceria nanoparticles was determined by a ferricytochrome C based assay.

## Results

The mean hydrodynamic diameter of PEG CeNPs in MillQ and culture medium were 250.6 nm and 153.3 nm respectively whereas Zeta potential were found to be -7.86 mV and -10.4 mV respectively. CeNPs did not exhibit cytotoixicity at the tested doses and time period in human epidermal cells (A431) as evident by MTT- and NR uptake- assays. The co-incubation of CeNPs with A431 cells showed significant (p<0.05) decrease in ROS generation as evident by a decrease in the DCFDA fluorescence.

## Conclusion

The present study demonstrates that CeNPs are stable in cell culture media conditions, non cytotoxic to A431 cells up to a relatively high concentration. CeNPs show free radical scavenging activity when co-incubated with A431 cells.

